# Assessing the predictive value of common gait measure for predicting falls in patients presenting with suspected normal pressure hydrocephalus

**DOI:** 10.1186/s12883-021-02068-0

**Published:** 2021-02-08

**Authors:** Alexander Davis, Mark Luciano, Abhay Moghekar, Sevil Yasar

**Affiliations:** 1 Department of Neurology, Johns Hopkins University School of Medicine, 5200 Eastern Ave, Center Tower, STE 5100, Baltimore, MD 21224 USA; 2 Department of Medicine, Johns Hopkins University School of Medicine, Baltimore, MD USA

**Keywords:** Normal pressure hydrocephalus, Fall risk, Falls, Timed up & go, Fall prediction, Fall risk Questionnare

## Abstract

**Objective:**

To assess the predictive value of common measures validated to predict falls in other geriatric populations in patients presenting with suspected Normal Pressure Hydrocephalus (NPH).

**Methods:**

One hundred ninety-five patients over the age of 60 who received the Fall Risk Questionnaire were retrospectively recruited from the CSF Disorders clinic within the departments of Neurosurgery and Neurology. Multiple logistic regression was used to create a model to predict falls for patients with suspected NPH using common measures: Timed Up & Go, Dual Timed Up & Go, 10 Meter Walk, MiniBESTest, 6-Minute Walk, Lower Extremity Function (Mobility), Fall Risk Questionnaire, and Functional Activities Questionnaire.

**Results:**

The Fall Risk Questionnaire and age were shown to be the best predictors of falls. The model was 95.92% (Positive predictive value: 83.93%) sensitive and 47.92% specific (Negative predictive value: 77.78%).

**Conclusion:**

Patients presenting with suspected NPH are at an increased fall risk, 75% of the total patients and 89% of patients who responded to a temporary drain of CSF had at least one fall in the past 6 months. The Fall Risk Questionnaire and age were shown to be predictive of falls for patients with suspected NPH. The preliminary evidence indicates measures that have been validated to assess fall risk in other populations may not be valid for patients presenting with suspected NPH.

**Supplementary Information:**

The online version contains supplementary material available at 10.1186/s12883-021-02068-0.

## Background

Normal Pressure Hydrocephalus (NPH) is defined by the triad of gait disturbances, cognitive decline, and urinary incontinence; coinciding with enlarged communicating ventricles [1]. Gait disturbance is an essential symptom within the triad with at least one of the other two symptoms present. The hallmark gait disturbance of NPH is a slow broad-based gait, with outwardly rotated feet, and a diminished step height (shuffle) [2].

To assess a patient’s fall risk subjective questionnaires, and objective clinical tests are the most common measures used. Numerous subjective questionnaires and objective clinical tests have been validated to predict falls in geriatric patients with neurological conditions [3–5]. However, the use of these measures to predict falls has not been validated for use in patients with suspected NPH. With NPH having a unique gait disturbance it is plausible that the cause of falls could be different from other neurological conditions [1].

The literature is sparse regarding which objective or subjective measures or combination of measures are predictive of falls in patients presenting with suspected NPH. The present study aims to address this lack of information by assessing the predict value of measures validated to predict falls in other populations. We hypothesize that we will identify a measure or combination of measures that is sensitive and specific, predicting which patients will have had at least one fall in the past 6 months.

## Methods

### Participants

One hundred ninety-five patients over the age of 60 presenting with suspected NPH who received the Fall Risk Questionnaire were retrospectively recruited from the CSF Disorders clinic within the departments of Neurosurgery and Neurology, between June 2016 and April 2019. Patients were considered to have suspected NPH if they presented with at least two symptoms in the NPH (gait disturbances, cognitive decline, and urinary incontinence) coinciding with enlarged communicating ventricles, without antecedent causes. This study was approved by the Johns Hopkins IRB: Cerebrospinal Fluid Disorders Biorepository & Adult Hydrocephalus Clinical Research Network NA_00029413. All study protocols followed the guidlnies set forth by the Johns Hopkins IRB. The study being a retrospective study involving only data extraction and analysis, informed consent was waived by the IRB. Data once extracted was anonymized for analysis.

### Measures

Assessments were completed in a clinical setting by either a physical therapist or a research assistant.

#### Gait velocity

Patients were instructed to walk “As quickly as you can safely.” for all gait velocity measures. Ten meter Walk Test (10MWT); patients walked ten meters in a straight line [6]. Timed Up & Go (TUG) patients started seated in a chair with armrests. Walked ten feet, turned 180 degrees, walked back to the chair, and sat down [7]. Dual TUG Is identical to the TUG except while patients walked they perform a serial subtraction of the number three [8]. Gait velocity measured using time to complete, with lower times indicating better peroformance.

#### Endurance

6-Minute Walk Test (6MWT): Patients walked as far as they could in 6 min [9]. The score for the 6MWT was total distance walked in feet, with greater distance indicating better performance.

#### Balance

Mini-Balance Evaluation Systems test (Mini-BESTest) is a dynamic measure of balance consisting of fourteen items all scored from 0 to 2. The maximum score was 28 points with a higher score indicating better balance [10].

### Subjective questionnaires

From the Quality of Life in Neurological Disorders (Neuro-QoLv1.0), Lower Extremity Function (Mobility) short form (SF-LEF); measures patient self-reported functional mobility lower scores indicate lower functional mobility [11]. Fall Risk Questionnaire (FRQ) is a clinically validated, self-rated subjective fall screening test [5]. For the Fall Risk Questionnaire, to attempt to better predict falls within the NPH population four questions were added that are pertinent to the gait of patients with NPH; “I have difficulty walking on uneven surfaces”, “I have difficulty initiating gait or I tend to freeze while walking”, “I tend to shuffle my feet while walking” and “I have difficulty turning while walking” higher scores indicate a greater fall risk. Functional Activities Questionnaire (FAQ) is an informant rated measure using a scale assessing instrumental activities of daily living (IASDLs) higher scores indicate impairment in IADLs [12].

### Outcome measure

From the FRQ, the question “I have fallen in the last 6 months. “Yes, or No” was used to determine if the patient had experienced a fall in the last 6 months (removed from the FRQ before analysis). The 6 month time frame for fall assement is consistent with other validated fall prediction measures [13].

### Statistical analysis

Patient baseline characteristics were summarized using frequencies with percentages or means with standard deviations (SDs). Analyses were performed using STATA version 15.1 (Stata Corp LP, Inc., College Station, TX). All reported *p* values are two-sided, and significance was set at *p* < 0.05. The question “I have fallen in the last 6 months.” was removed from the FRQ before analysis due to it being a direct measure of the outcome variable. Log conversions were used to correct skewed data [14].

The goal of the analysis was to create a retrospective model to predict which patients presenting with suspected NPH have had at least one fall in the past 6 months. The number of patients who were referred for shunt surgery was not large enough to compute a separate analysis. First, a correlation matrix and frequencies tables were created to assess patterns in the data. Multiple logistic regression was used to create the fall risk model, all nine measures were regressed unadjusted, and Receiver Operator Characteristic (ROC) curves were calculated. All nine measures were run stepwise, adding in relevant control variables at each stage. Lastly, all combinations of measures that were trending or significant were analyzed using forward stepwise variable selection [15]. The confounding variables were; age, sex, past medical history affecting gait (cerebral vascular accident, transient ischemic attack, Parkinson’s disease, meningioma, spinal disorders, degenerative joint disease, neuropathy, and osteoarthritis), and depression medication.

## Results

Table 1 shows patient characteristics by fall status.

**Table 1:** Baseline demographics, clinical characteristics of (*N* = 195) study patients and subset of the patients with and without a fall in the past 6 months

	All Patients Mean (SD) N (%) *N* = 195	No Fall Mean (SD) N (%) *N* = 48	Fall Mean (SD) N (%) *N* = 147	p
Age (years)	75.13 (6.5)	72.71 (7.54)	75.94 (5.92)	0.003
Sex (male)	116 (59.49)	31 (64.58)	85 (57.82)	
Race (Caucasians)	173 (88.72)	41 (85.42)	132 (89.8)	
Education (years)				
≤ 12	59 (31.05)	18 (38.3)	41 (28.67)	
13–16	70 (36.84)	16 (34.04)	54 (37.76)	
> 16	61 (32.11)	13 (27.66)	48 (33.57)	
EVANs Index (EI)	0.37 (0.04)	0.37 (0.37)	0.37 (0.04)	
Height (meters)	1.69 (0.1)	1.71 (0.11)	1.68 (0.01)	
Body Mass Index (BMI)	27.81 (4.58)	27.26 (4.51)	27.99 (4.6)	
Assistive Device				0.011
None	79 (40.51)	27 (56.25)	52 (35.37)	
Cane	49 (25.13)	14 (29.17)	35 (23.81)	
Walker	67 (34.36)	7 (14.58)	60 (40.82)	
Past Medical History (positive %)	107 (54.87)	26 (54.17)	81 (55.1)	
FRQ	7.87 (2.54)	5.67 (2.90)	8.59 (1.94)	< 0.001
NPH FRQ	10.83 (3.43)	8.15 (4.02)	11.71 (2.71)	< 0.001
SF-Mobility	27.34 (8.47)	32.09 (7.44)	25.82 (8.24)	< 0.001
TUG	24.82 (24.91)	17.37 (11.40)	27.25 (27.54)	
Dual TUG	34.61 (28.43)	24.43 (19.85)	37.84 (42.22)	0.014
10 MWT	18.66 (21.96)	12.10 (6.36)	20.82 (24.69)	0.032
MiniBESTest	14.69 (4.99)	16.33 (4.87)	14.16 (4.94)	0.010
6 MWT	800.01 (436.13)	956.43 (428.61)	747.50 (427.39)	0.004

Past Medical History positive for any of the following: stroke, transient ischemic attack, Parkinson’s disease, brain tumor, spinal disorders, degenerative joint disease, neuropathy or osteoarthritis.

*FRQ* Fall Risk Questionnaire, *NPH FRQ* NPH Fall Risk Questionnaire, *SF-Mobility* Short Form–Lower Extremity Function, *MoCA* Montreal Cognitive Assessment, *SDMT* Symbol Digit Modalities Test, *TUG* Timed Up & Go, *Dual TUG* Dual Timed Up & Go, *10 MWT* 10 Meter Walk test, *Mini-BEST* Mini-Balance Evaluation Systems test, *6 MWT* 6-Minute Walk test

Patients on average are approximately 75 years of age, predominately Caucasian, highly educated, and overweight. Patients who have had at least one fall in the past 6 months on average were older and more impaired than patients that did not experience a fall.

Table 2 shows the output of the fall prediction model including adjusted odds ratios with 95% confidence intervals, and *p* values.

**Table 2:** Logistic regression analysis for patients presenting with suspected NPH

Variables	Adjusted OR (CI 95%)	*P*-value
Fall Risk Questionnaire	1.43 (1.18–1.72)	< 0.001
Age	1.09 (1.02–1.15)	0.006
Assistive Device		
None	1	
Cane	0.38 (0.14–1.07)	0.067
Walker	1.01 (0.31–3.35)	0.982

Not included in the table because not significant: Sex and Past Medical History Affecting Gait

Sensitivity: 95.92%

Specificity: 43.75%

Positive predictive value: 83.93%

Negative predictive value: 77.78%

Correctly classified: 83.08%

**Fig. 1:**
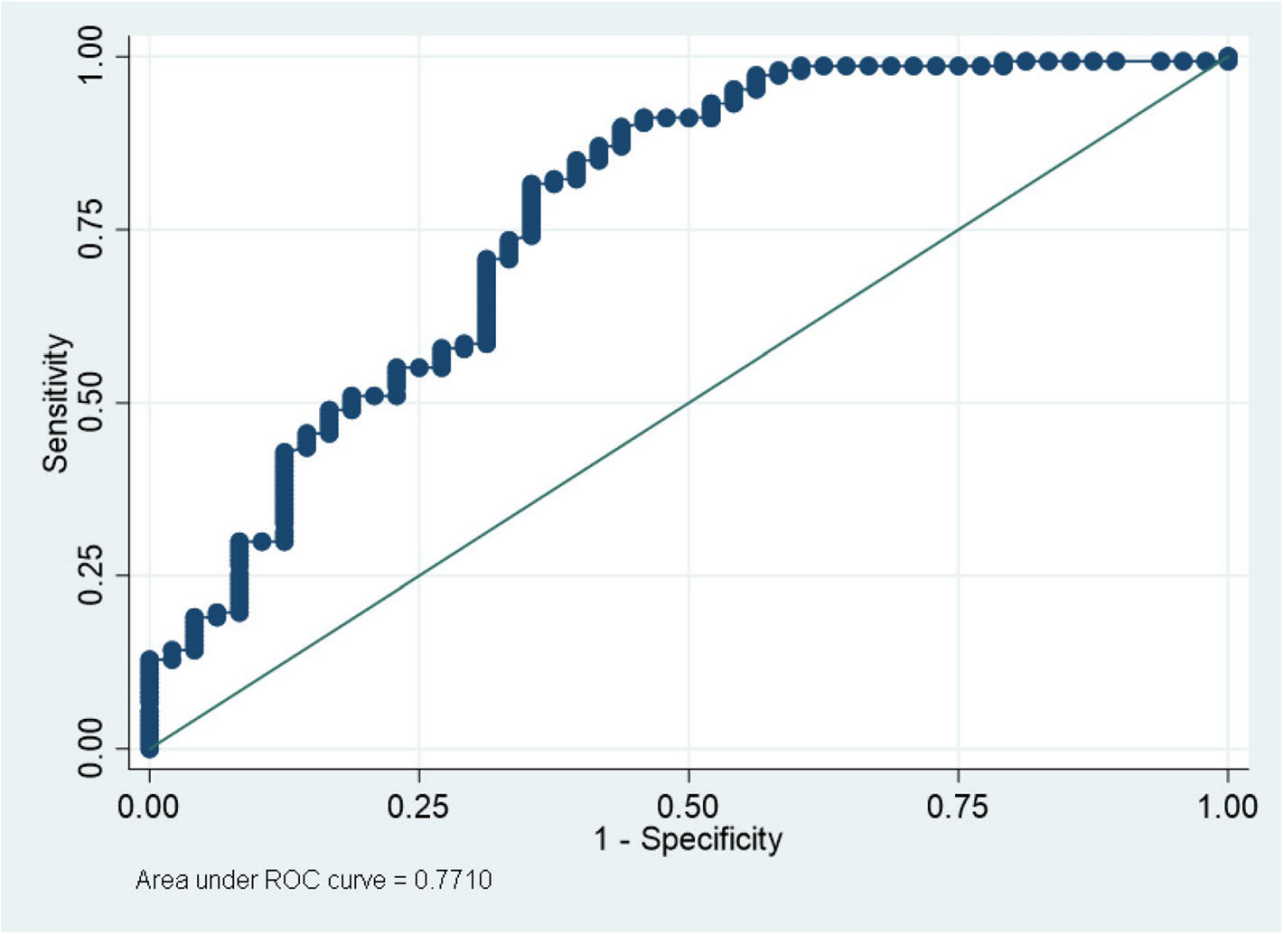
ROC curve for Fall Prediction Model

The area under the ROC curve for the fall prediction model is 0.771 (Fig. 1).

The model comprises 195 patients, 147 patients had at least one fall in the past 6 months. For those patients, the model correctly identified 141 cases (Sensitivity: 95.92%, Positive predictive value: 83.93%). For the 48 patients that did not experience a fall within the past 6 months, the model correctly predicted 23 cases (Specificity: 47.92%, Negative predictive value: 77.78%).

The FRQ was the only measure when adjusted to significantly predict falls for patients presenting with NPH in our dataset. Age was a significant factor in predicting falls with older patients being more likely to have fallen. As well, assistive device use was trending, with patients who used a can being less likely to fall than patients who did not. The NPH Fall risk Questionnaire performed identically to the original version, parsimony indicates the original version is a better measure for predicting falls in the suspected NPH population.

## Discussion

The fall risk model was significantly predictive of which patients will have at least one fall in the past 6 months (95.92% sensitivity, positive predictive value: 83.93, 43.75% specificity, negative predictive value: 77.78%). Presently, there are only studies assessing fall risk in healthy elderly patients or in other neurological conditions such as Parkinson’s disease [3–5]. There are no studies of patients presenting with suspected NPH to compare our results to. The FRQ was the only measure significant when adjusted. Age was also predictive of falls, with older patients being more likely to experience a fall. The NPH FRQ did not add any predictive value over the original version. The original version of the FRQ requires fewer items; therefore, it is the better measure due to parsimony.

Annually, 35 to 40% of community-dwelling adults over the age of 65 experience at least one fall [16]. In contrast, 75% of patients with suspected NPH have experienced at least one fall in the past 6 months. As well, in our data we found that 89% of the patients that significant improvement in gait following temporary drainage of CSF have experienced at least one fall in the past 6 months. The increased prevalence of falls coinciding with the potential for serious injury shows the need to identify the best measure(s) to assess the fall risk of patients presenting with suspected NPH [17]. The preliminary evidence in this study shows that measures validated to assess fall risk in other similar populations may not be valid for patients with NPH.

For the clinical application of this model, we found that 89% of patients who had experienced significant improvement in their gait following temporary drainage of CSF had at least one fall in the previous 6 months. Due to the extremely high prevalence of falls in patients who respond, we recommend assuming all patients referred for shunt surgery are at a significant fall risk. We have a subsequent study planned to assess falls in the time between shunt referral and shunt placement.

Quantifying the outcome of shunt surgery has been a primary interest of NPH researchers since the condition was discovered [18]. Researchers have used many different approaches to determine if a patient has improved after they were shunted, including identifying if shunted patients have a reduced fall risk compared to before they were treated. The logical assumption that improvement on measures shown to be predictive of falls in multiple other similar populations would lead to a decreased risk of falling is contradicted by the preliminary evidence shown in this study.

This studies limitations include the need for the fall risk model to be validated in a second prospective study. With the objective and subjective measures being performed after the 6 month period where falls were measured there is potential for decline in symptoms to misrepresent the actual level of impairment for when falls were measured. As well, we did not have data on vision or hearing impairment or medication use that could affect falls. The strengths of our study includes a large number of participants with detailed and well established quantitative and subjective measures. There is a subsequent study planned to validate the fall risk model using prospective data collection.

## Conclusion

In this study we created a fall risk model that was significantly predictive of which patients will have at least one fall in the past 6 months (95.92% sensitivity, Positive predictive value: 83.93, 43.75% specificity, Negative predictive value: 77.78%). Patients presenting with suspected NPH are at an increased fall risk, 75% of the total patients and 89% of patients who responded to a temporary drain of CSF had at least one fall in the past 6 months. In creating this model we found that measures that have been validated to predict falls in other populations were unable to predict falls in the suspected NPH population.

## Supplementary Information

**Additional file 1.** Normal Pressure Hydrocephalus – Fall Risk Questionnaire.

## Data Availability

Anonymized raw data will be made available upon reasonable request.
