# Supplementary material for: Assessing the predictive value of common gait measure for predicting falls in patients presenting with suspected normal pressure hydrocephalus
Source: BMC Neurol. 2021 Feb 8;21:60. doi: 10.1186/s12883-021-02068-0 (PMC7869204; doi:10.1186/s12883-021-02068-0)
Supplement: Supplementary file 1 — Additional file 1. Normal Pressure Hydrocephalus – Fall Risk Questionnaire. [file 12883_2021_2068_MOESM1_ESM.docx]

Assessing the Predictive Value of Common Gait Measure for Predicting Falls in Patients Presenting With Suspected Normal Pressure Hydrocephalus

Authors: Alexander Davis, BA; Mark Luciano, MD, PhD; Abhay Moghekar, M.B.B.S; Sevil Yasar, MD, PhD

Alexander Davis, Johns Hopkins University School of Medicine, Department of Neurology, Baltimore, MD, USA

Mark Luciano, Johns Hopkins University School of Medicine, Department of Neurosurgery, Baltimore, MD, USA

Abhay Moghekar, Johns Hopkins University School of Medicine, Department of Neurology, Baltimore, MD, USA

Sevil Yasar, ^3^Johns Hopkins University School of Medicine, Department of Medicine, Baltimore, MD, USA

| Normal Pressure Hydrocephalus – Fall Risk Questionnaire | | |
| --- | --- | --- |
| Please answer the following questions: | Yes | No |
| I have fallen in the last 6 months |  |  |
| I use or have been advised to use a cane or walker to get around safely |  |  |
| I am worried about falling |  |  |
| Sometimes, I feel unsteady when I am walking |  |  |
| I steady myself by holding onto furniture when walking at home |  |  |
| I need to push with my hands to stand up from a chair |  |  |
| I have some trouble stepping up onto a curb |  |  |
| I often have to rush to the toilet |  |  |
| I have lost some feeling in my feet |  |  |
| I take medicine that sometimes makes me feel light-headed or more tired than usual |  |  |
| I take medicine to help me sleep or improve my mood |  |  |
| I often feel sad or depressed |  |  |
| I have difficulty walking on uneven surfaces |  |  |
| I have difficulty initiating gait or I tend to freeze while walking |  |  |
| I tend to shuffle my feet while walking |  |  |
| I have difficulty turning while walking |  |  |
